# Spatial and Temporal Expression of High-Mobility-Group Nucleosome-Binding (HMGN) Genes in Brain Areas Associated with Cognition in Individuals with Down Syndrome

**DOI:** 10.3390/genes12122000

**Published:** 2021-12-17

**Authors:** Alejandra Rodríguez-Ortiz, Julio César Montoya-Villegas, Felipe García-Vallejo, Yecid Mina-Paz

**Affiliations:** 1 Laboratory of Molecular Biology and Pathogenesis, Department of Physiological Sciences, School of Basic Sciences, Faculty of Health, Universidad del Valle, Cali 76001, Colombia; alejandra.rodriguez@correounivalle.edu.co (A.R.-O.); julio.montoya@correounivalle.edu.co (J.C.M.-V.); jesus.garcia@correounivalle.edu.co (F.G.-V.); 2 Health and Movement Research Group, Faculty of Health, Universidad Santiago de Cali, Cali 76001, Colombia

**Keywords:** Down Syndrome, gene expression, epigenetics, human brain, high-mobility-group N proteins (HMGN), neuroinformatics

## Abstract

DNA methylation and histone posttranslational modifications are epigenetics processes that contribute to neurophenotype of Down Syndrome (DS). Previous reports present strong evidence that nonhistone high-mobility-group N proteins (HMGN) are epigenetic regulators. They play important functions in various process to maintain homeostasis in the brain. We aimed to analyze the differential expression of five human HMGN genes in some brain structures and age ranks from DS postmortem brain samples. **Methodology:** We performed a computational analysis of the expression of human HMGN from the data of a DNA microarray experiment (GEO database ID GSE59630). Using the transformed log2 data, we analyzed the differential expression of five HMGN genes in several brain areas associated with cognition in patients with DS. Moreover, using information from different genome databases, we explored the co-expression and protein interactions of HMNGs with the histones of nucleosome core particle and linker H1 histone. **Results:** We registered that HMGN1 and HMGN5 were significantly overexpressed in the hippocampus and areas of prefrontal cortex including DFC, OFC, and VFC of DS patients. Age-rank comparisons between euploid control and DS individuals showed that HMGN2 and HMGN4 were overexpressed in the DS brain at 16 to 22 gestation weeks. From the BioGRID database, we registered high interaction scores of HMGN2 and HMGN4 with Hist1H1A and Hist1H3A. **Conclusions:** Overall, our results give strong evidence to propose that DS would be an epigenetics-based aneuploidy. Remodeling brain chromatin by HMGN1 and HMGN5 would be an essential pathway in the modification of brain homeostasis in DS.

## 1. Introduction

The continuous chromatin modification and the binding of tissue-specific transcription factors to their specific targets in chromatin maintain the epigenetic landscape necessary to regulate the cell-type-specific transcription [1,2]. However, additional chromatin modifiers, including the H1 linker histones [2] and the high mobility group N (HMGN) proteins, can remodel the chromatin organization and transcription regulation, playing important functions in several process to maintain the general homeostasis [3]. Down Syndrome (DS) is a chromosomal aneuploidy caused by a total or partial triplication of chromosome 21, but in rare cases it can be associated with a process of chromosome translocation [4]. In people with DS, the gene dose imbalance by triplication of genes on HSA21 is mostly associated with a wide spectrum of pathologies that include neurological and systemic diseases [4]. The incidence of trisomy 21 is influenced by maternal age and differs throughout the population [5,6]. In developed countries, the average life span for DS population is 55 years [7]. Although DS is the result of the increased copy number of a single 21 chromosome, the regulation of gene expression is affected at a genome-wide level [8,9,10,11].

There is a growing line of evidence proposing that beyond HSA21 trisomy, DS is an epigenetics-based syndrome [12]. For instance, a previous study with fetal skin fibroblasts from a set of monozygotic twins revealed regions that were predominantly hypermethylated in DS in genes involved in embryonic organ morphogenesis. Reprogramming of the DS fibroblasts to induced pluripotent stem cells (iPSCs) showed that these regions were maintained in the pluripotent state and correlated with differential gene expression and increased expression of the DNA methyltransferases *DNMT3B* and *DNMT3L* [13]. Thus, the genome-wide differences seen in DS tissues are correlated with epigenetic modifications that would be responsible, in part, for the establishment and/or maintenance of differential expression of genes in and outside of HSA21 in DS.

HMGN proteins are a nonhistone protein family that includes five members encoded by five specific genes with a similar intron-exon organization, localized along human genomes in different chromosome loci (Table 1) [13,14,15,16,17,18]. Previous reports show that HMGN proteins are the only nuclear proteins known to specifically recognize the generic structural features of the 147 base pair nucleosome core particles. In vitro analyses showed that at low ionic strength, nucleosome core particles can bind to HMGN proteins with high affinity [18,19,20,21]. The interaction between HMGN proteins and nucleosomes is dynamic, and the proteins compete among themselves and with the linker histone H1 for chromatin binding sites [22,23,24]; in fact, all HMGN proteins have similar affinities when binding to chromatin [22]. This interaction has been shown to affect the compaction level of the chromatin, modulating epigenetic events, and defining transcription profiles [21].

Since HMGN proteins play an important role as molecules that reshape the organization of chromatin and transcription levels, we performed a bioinformatic simulation analysis of their spatial and temporal expression in several areas of the brain with DS. To perform these analyses, we obtained log2 data from a free-access microarray previously consigned in the GEO DataSets of NCBI (https://www.ncbi.nlm.nih.gov/ (accessed on 1 December 2018). To explore the differential brain expression of HMGN genes, we calculated the *Z*-ratio from DS postmortem brain samples, specifically from those brain areas associated with cognitive processes previously described by Olmos-Serrano et al., 2016 [25].

## 2. Methodology

### 2.1. Data Mining

Raw gene expression data of DS samples and normal samples were downloaded from the Gene Expression Omnibus (GEO) (http://www.ncbi.nlm.nih.gov/geo/ (accessed on 1 December 2018)) of the National Center for Biotechnology Information (NCBI). For the analyses performed in the present study, we selected the human HMGN genes previously consigned in the Gene Entrez of the NCBI database (https://www.ncbi.nlm.nih.gov/gene (accessed on 17 September 2020)) (Table 1)**.** Moreover, for all calculations, we used the log2 transformed expression values of free-access DNA microarray experiment whose registration code in the GEO database is GSE59630 (http://www.ncbi.nlm.nih.gov/geo/query/acc.cgi?acc=GSE59630 (accessed on 1 December 2018)), previously deposited by Olmos-Serrano et al. 2016. [25].

According to the information consigned in the GEO database, the selected microarray experiment included gene expression data of more than 17000 probes from 58 post-mortem brain samples of DS individuals (25 from females and 33 from males) and 58 euploid samples as euploid controls (25 from females and 33 from males) that were classified by sex, age, and also by some brain areas including the hippocampus (HIP), cerebellar cortex (CBC), dorsolateral prefrontal cortex (DFC), orbital prefrontal cortex (OFC), ventrolateral prefrontal cortex (VFC), medial prefrontal cortex (MFC), primary somatosensory cortex (S1C), inferior parietal cortex (IPC), primary visual cortex (V1C), superior temporal cortex (STC), and inferior temporal cortex (ITC). Nevertheless, for the present study, we decided to analyze, not only the brain as a whole, but also OFC, MFC, HIP, and CBC brain regions that are highly associated with neurophenotypes of DS.

### 2.2. Data Preprocessing

Using the Partek Genomics Suite version 6.7 (Partek Incorporated, St. Louis, MO, USA), the robust multiarray analysis (RMA) algorithm [26] in Affymetrix Power Tools (APT; http://www.affymetrix.com/, accessed on 1 December 2018) was applied by Olmos-Serrano et al. 2016. [25], combined with an R-script to perform background correction and standardization for all raw data, aiming to filter false-positive data. The applied criterion was as follows: at least half the samples had PLIER signal intensity values greater than 100 [27].

### 2.3. Quantification of the Differential HMGN Genes Expression

Raw intensity log2 data from each experiment were used for the calculation of *Z*-score [28]. *Z*-scores of the protein coding genes analyzed were calculated according to Equation (1):

(1) $$Z-score=\frac{\left(Log\;intensity\;of\;G-mean\log intensity\;G\ldots Gn\right)}{Standard\;Deviation\log G\ldots Gn}$$

Equation (1). *Z*-score formula

All *Z*-score values were normalized on a linear scale −3.0 ≤ 0 ≥ +3.0 (two-tailed *p* value < 0.001). From *Z*-score data, we calculated the mean values per gene and per structure in brain samples of *DS* and euploid controls. These data were used to calculate the *Z*-ratio (Equation (2)), a measure to estimate differential gene expression, where genes with values over 1.96 are considered over-expressed [28].

(2) $$Z-ratio=\frac{\left[\left(Z-score_{G1ave}\right)DS-\left(Z-score_{G1ave}\right)Con\right]}{SD\;of\;Z-score\;differences_{G1\ldots Gn}}$$

Equation (2). *Z*-ratio formula

### 2.4. Gene-Dosage Imbalanced Quantification

To find out the gene dosage imbalance of the five HMGN genes in the structures of DS brain samples, first we calculated the *M* values according to Equation (3), and then we used the *M* value to calculate the ratio of the dosage imbalance *R* (*DS Control* ratio) as shown in Equations (3) and (4) [29].

Equation (3) *M*-value formula

(3) $$M=[Mean\;Log_{2}\left(DS\right)-Mean\;Log_{2}\left(Control\right)$$

Equation (4) *R* (*DS*/*Control* ratio) formula

(4) $$R=2^{M}$$

*R* values ranging from 0.80 to 1.30 were considered as normal balanced (two copies of the gene); on the contrary, if *R* values were in the range 1.4 ≤ 1.5 ≥ 1.7, genes were dosage-imbalanced by triplication (three copies per gene), but if *R* ratio was greater than 1.8, genes were amplified (more than three copies).

### 2.5. Construction of HMGN Genes Network Using GeneMania

To build the gene interaction network of HMGN genes, we used the free-access platform GeneMANIA (http://www.genemania.org (accessed on 17 September 2020)), a real-time multiple association network actively developed at the University of Toronto, in the Donnelly Centre for Cellular and Biomolecular Research that uses a massive set of functional association data [30]. All calculations carried out in the present study were processed using the updated 2018 version [31].

### 2.6. Protein–Protein Interaction Analysis

To simulate the interaction between each HMGN with several histones of the core particle and H1, we obtained data from BioGRID (Database of Protein, Chemical, and Genetic Interactions), a free-access database (https://thebiogrid.org/ (accessed on 17 September 2020)) [32,33]. BioGRID is an interaction repository with data compiled through comprehensive curation efforts. The current index is version 3.5 and all data are freely provided via their search index and available for download in standardized formats. The different searches performed in the present study, were from data updated by January of 2019 [32,33].

### 2.7. Statistical Analysis

To compare mean values of *Z*-ratio of DS brain, we performed multivariate statistical analyses among the different brain cortex structures between DS patients and euploid controls. The *Z* test/Two-tailed was used to calculate differences in HMGN differential expression. The p-values were calculated using the web tool *p*-value from *Z*-score Calculator (https://www.socscistatistics.com/pvalues/normaldistribution.aspx (accessed on 17 September 2020)). In all cases, we used an alpha of 0.05 to test the significance of H_0_. All analyses were run in SPSS program version 25.0 (https://spss.softonic.com/ (accessed on 17 September 2020)) and Cytoscape 3.6 (https://cytoscape.org/release_notes_3_6_0.html (accessed on 17 September 2020)).

## 3. Results

### 3.1. Expression of HMGN Genes in Brain Areas from Individuals with DS

In general, we observed that the expression of five HMGN genes was variable along all structures under analysis. Moreover, we recorded significant differences in their overexpression values depending of the brain area under analysis. In this sense, the genes encoding for HMGN1 and HMGN5 were overexpressed not only in HIP, CBC, and V1C but also in some areas of prefrontal cortex including DFC, OFC, and VFC (values of *Z*-ratio > 1.96); in contrast, *HMGN2* and *HMGN3* genes had not significant overexpression. Only in ITC did the *HMGN5* gene register a significant *Z*-ratio (*Z*-ratio = 2.0) (Table 2).

Since the *HMGN1* gene is localized at the 21q22.2 band, we calculated the level of dose imbalance in those brain structures including in the present study. Our results showed that *HMGN1* was dosage imbalanced, in OFC, VFC, and CBC by triplication (R > 1.4), but in HIP, DFC, and ITC, it was dysbalanced by amplification (R > 1.8).

### 3.2. Age Dependent Expression of HMGN Genes in the Brain of Individuals with DS

*HMGN4* (*Z*-ratio = 4.72) and *HMGN2* (*Z*-ratio = 2.13) were significantly overexpressed in prenatal samples of DS brain (16 to 22 weeks of gestation) in comparison to other age ranks (Table 3). *Z*-ratio data for *HMGN3* showed significant overexpression values in the brain of DS during the first year (0-12 months), childhood (2 to 10 years), 12- to 22-year-old samples, and adulthood (32 to 42 years old), but not in brain samples of prenatal brain samples (16 to 22 weeks of gestation; *Z*-ratio = 1.08) (Table 3). In contrast, *Z*-ratio values for *HMGN1* and HMGN5 along the different age ranks were non-significant, except for *HMGN1* that was overexpressed in rank of 12 to 22 years old (*Z*-ratio = 2.0) (Table 3).

### 3.3. Protein–Protein Interaction Network and GO Categories

**Figure 1:**
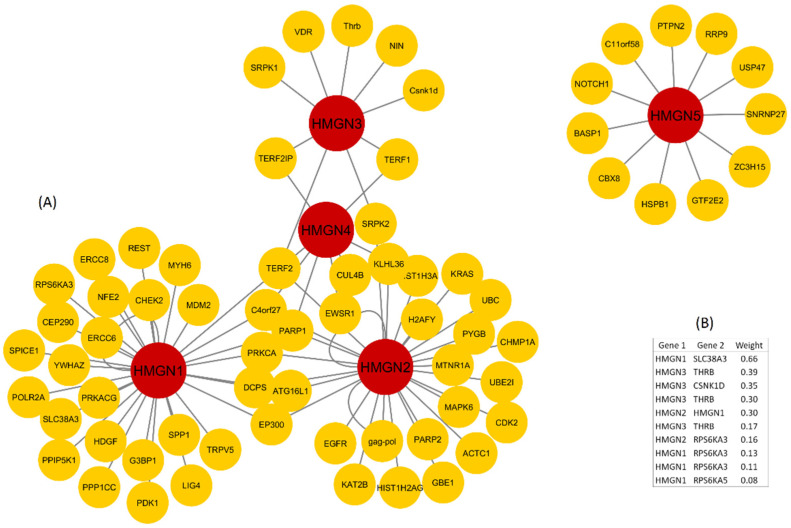
Protein-to-protein interaction network between the five human High-Mobility-Group Nucleosome-Binding (HMGN) proteins and several cellular human genes. (**A**) Topological structure of the network using Cytoscape program. (**B**) The statistically significant cellular genes that interact with some of one HMGN. Data were obtained from GeneMania database.

The Protein–Protein Interaction (PPI) network made with the five HMGN genes accounted for a total of 73 nodes, two connected components, one multi-edge node pair, an average number of neighbors of 2.374, and a heterogeneity of 2.012 (Figure 1). The node with the highest number of interactions was HMGN1 with 30, followed closely by HMGN2 with 28. Most relevant GO categories of biological processes obtained from the network included indispensable epigenetic processes for chromatin activation or inactivation such as histone deacetylation (*p*-value 2.46 × 10^−8^) and histone H3-K4 methylation (*p*-value 1.88 × 10^−7^).

### 3.4. HMGN Protein Interaction with Histones of Nucleosome Core and Linker H1

Data from several experimental methods reported in BioGRID database showed differential high interaction scores of HMGN proteins with histones of the nucleosome core HIST1H2AG, HIST1H2BA, HIST1H3A, and HIST1H4A (Table 4). Only HMGN2 had a significant high score of interaction with the linker histone HIST1H1A and the histones of nucleosome core HIST1H3A and HIST1H2AG. HMGN1 and HMGN5 showed significant interaction scores only with HIST1H4A. Finally, HMGN3 interacts with the HIST1H4A.

## 4. Discussion

Previously, some studies presented strong evidence that in DS individuals the genome-wide epigenomic alterations occur not only in chromosome 21 but also in some other chromosomes [34,35,36]. These include changes in gene expression, RNA content, and epigenetic histone modifications, nucleosome spacing, and DNA methylation process, which are dependent on health status and age [12]. Specifically, there is strong evidence that HMGN proteins play a role in epigenetic regulation of gene expression and play important functions in several biological processes to maintain normal homeostasis and altered gene expression in disease [12,37,38]. Thus, we aimed to analyze, using a bioinformatics approach, the gene expression of human HMGN genes in different human brain structures and age ranks, comparing DS brain samples and euploid controls.

Most of the literature reports about the expression of HMGNs in the brain as as whole and also in neuronal derived cells come from experiments carried out in mice [39]. In this sense, its interpretation could be cautiously extended to the brain of individuals with DS. In this scenario, our results are the first to analyze, in a representative sample of euploid individuals and DS individuals, the differential expression of the five human HMGNs in several area of the brain that are involved in learning and memory and also its age rank variation. Our bioinformatics approach allowed us to obtain strong statistical evidence of the differential regulation of HMGNs in the disruption of the normal brain homeostasis in some areas associated with the DS neurophenotype.

*HMGN1* is in a region of human chromosome 21, and it is frequently found triplicated in DS samples. Our results not only confirmed the previous reports but extended the data to the brains of individuals with DS. The HMGN1 gene was dose-dysbalanced by triplication in the whole brain and brain cortex and dysbalanced by amplification in the hippocampus. The hippocampus is a brain structure that plays a major role in neural plasticity and cognition [40], which is known to the dysregulated in individuals with DS. Our results showed that methyl CpG-binding protein 2 (*MeCP2*) is underexpressed in several structures of the brain of DS, which can be linked to the dysregulation of the *HMGN1* gene, given that this latter gene can affect the expression of *MeCP2* by changing the chromatin structure and histone modifications in the *MeCP2* promoter [40].

According to the PPI network, the nodes with the highest number of interactions were HMGN1 and HMGN2. Moreover, the GO categories showed a global implication of these genes in chromatin remodeling processes such as acetylation, methylation of histones, and dendritic spine morphogenesis. The dysregulation of gene expression recorded in these genes would most certainly affect the interactions with others and would possibly lead to the epigenomic changes found in individuals with DS.

Some analysis suggests that HMGNs could differentially modulate the global gene transcription in not only some brain structures but also in other tissues [40]. Therefore, the contribution of *HMGN1* and *HMGN5* to the transcriptional dysregulation of DS neurophenotype needs to be studied separately in specific developmental scenarios [41]. In mice, HMGN1 is a negative regulator of the brain expression of *MeCP2*, which promotes *HMGN1* overexpression associated with some effects not only in general behavioral activities but also in anxiety and social deficits [38]. On the other hand, HMGN5, is thought to reduce the compaction of the chromatin fiber nucleosomes, thereby enhancing transcription from chromatin templates; nevertheless, it has not been related to DS.

Excluding the gestational period (12–16 WG), only *HMGN3* had a statistically significant expression across different age-ranks. It contrasts with the expression of *HMGN2* and *HMGN4*, which had significant differential expression in the gestational period but not in the rest of the age-ranks we evaluated. In this sense, our results support some data found in the literature that HMGN3 control part some epigenetic mechanisms during the neuronal development [42]. *HMGN2* expression has been widely associated with embryogenesis [43]; anti-sense manipulation of *HMGN2* gene leads to early embryonic abnormalities [44,45]. Our results suggest that HMGN2 regulates active and bivalent genes by promoting an epigenetic landscape of active histone modifications at promoters and enhancers, stabilizing the epigenetic landscape necessary to maintain the pluripotent identity of pluripotent stem cells [45].

## 5. Conclusions

Our results gave strong evidence to support the hypothesis of the crucial role of non-histones HMGN1 and HMGN5 proteins as important spatial and temporal remodelers that would change, by epigenetic process, the brain proteostasis in patients with DS. It is important to highlight that even though not all HMGN genes are located in chromosome 21, they presented a distinctive dysregulation, showing that the complexity seen in DS goes beyond chromosome 21. We also report the differential interaction of HMGN family proteins with histones of the nucleosome core HIST1H2AG, HIST1H4A, HIST1H2AG, HIST1H3A, HIST2H2AB, and HIST1H4A and also with the linker HIST1H1A. In this context, we propose that HMGN proteins play an important role in the topological process of remodeling the chromatin in several brain areas of individuals with DS that are associated with memory and learning processes. The global effect of this epigenetic deregulation would be the alteration of the brain homeostasis that potentially conditions the DS brain’s epigenetics mode.

## Figures and Tables

**Table 1:** Description of gene data of five encoding human high mobility binding nucleosome group proteins (HMGN). [Source of NCBI https://www.ncbi.nlm.nih.gov/gene/ (accessed on 17 September 2020)].

Gene	ID *	Locus	Gene Name
*HMGN1*	3150	21q22.2	High mobility group nucleosome binding domain 1
*HMGN2*	3151	1p36.11	High mobility group nucleosome binding domain 2
*HMGN3*	9324	6q14.1	High mobility group nucleosome binding domain 3
*HMGN4*	10473	6p22.2	High mobility group nucleosome binding domain 4
*HMGN5*	79366	Xq21.1	High mobility group nucleosome binding domain 5

(*). According to the classification of the National Center for Biotechnology Information (NCBI).

**Table 2:** Mean *Z*-ratio values of expression for the five human High-Mobility-Group Nucleosome-Binding (HMGN) genes in several brain structures of individuals with Down Syndrome (DS).

Gene	Gene ID	Brain	HIP	CBC	DFC	OFC	V1C	VFC	ITC
*HMGN1*	3150	2.81	3.00	2.35	2.63	2.07	3.00	1.86	2.45
*HMGN2*	3151	0.06	0.23	0.13	0.54	0.05	−0.62	0.68	0.99
*HMGN3*	9324	2.48	1.29	1.60	1.78	1.57	2.82	2.31	1.86
*HMGN4*	10473	0.37	0.85	0.77	0.27	0.84	0.41	0.19	−0.27
*HMGN5*	79366	2.65	2.18	1.60	2.49	1.13	1.49	2.53	2.88

*Z*-ratio value > 1.96 means significant gene overexpression in the brain of DS individuals. Gene ID and data source were obtained from the information consigned in NCBI GeoDataset of a microarray experiment with registration code of GSE59630. (HIP). Hippocampus; (CBC). Cerebellar brain cortex; (DFC). Dorsolateral prefrontal cortex; (OFC). Orbital prefrontal cortex; (V1C). Primary visual cortex (VFC). Ventrolateral prefrontal cortex and (ITC). Inferior temporal cortex.

**Table 3:** Mean values of *Z*-ratio for the five human High-Mobility-Group Nucleosome-Binding (HMGN) genes expressed in different age ranks of the brain of human Down Syndrome (DS) individuals.

Gene	16–22 WG	0–12 M	2–10 Y	12–22 Y	30–39 Y	40–42 Y
*HMGN1*	0.66	1.45	1.94	1.97	2.18	1.55
*HMGN2*	2.13	0.22	0.54	0.54	0.31	0.49
*HMGN3*	1.08	3.61	2.71	2.55	2.59	2.94
*HMGN4*	4.71	1.12	1.51	1.65	1.84	1.67
*HMGN5*	0.16	1.89	0.78	1.15	0.76	0.82

WG, Weeks of Gestation. M, Months. Y, Years. Data sources were previously consigned in the NCBI GeoDataset of a DNA microarray experiment under the registration code of GSE59630.

**Table 4:** BioGRID data of the five human HMGN interactions with the nucleosome core histones H2BA, H2AG, H2AB, H3A, and H4A and with the linker histone H1A.

Interactor	Interaction	Experimental Evidence	Throughput	Score *
HMGN1	HIST1H4A	Affinity Capture-MS (§)	High	>0.75
HMGN2	HIST1H2BA	Affinity Capture-MS	High	0.99
	HIST1H3A	Affinity Capture-MS	High	0.90
	HIST1H2AG	Affinity Capture-MS	High	0.77
	HIST1H1A	Proximity Label-MS (§§)	High	>0.75
HMGN3	HIST1H4A	Affinity Capture-MS	High	>0.75
HMGN4	HIST1H2AG	Affinity Capture-MS	High	0.92
	HIST1H3A	Proximity Label-MS	High	>0.75
	HIST2H2AB	Affinity Capture-MS	High	0.88
HMGN5	HIST1H4A	Affinity Capture-MS	High	>0.75

* The cut-off threshold is >0.75. Data from BioGRID (https://thebiogrid.org/ (accessed on 17 September 2020)). (**§**). Affinity Capture–MS interaction is inferred when a bait protein is affinity captured from cell extracts by either polyclonal antibody or epitope tag and the associated interaction partner is identified by mass spectrometric methods. (**§§**). Proximity Label–MS interaction is inferred when a bait-enzyme fusion protein selectively modifies a vicinal protein with a diffusible reactive product, followed by affinity capture of the modified protein and identification by mass spectrometric methods, such as the BioID system.

## Data Availability

DNA microarray experiment whose registration code in the GEO database is GSE59630 (http://www.ncbi.nlm.nih.gov/geo/query/acc.cgi?acc=GSE59630 (accessed on 1 December 2018)).
